# If these data could talk

**DOI:** 10.1038/sdata.2017.114

**Published:** 2017-09-05

**Authors:** Thomas Pasquier, Matthew K. Lau, Ana Trisovic, Emery R. Boose, Ben Couturier, Mercè Crosas, Aaron M. Ellison, Valerie Gibson, Chris R. Jones, Margo Seltzer

**Affiliations:** 1 School of Engineering and Applied Sciences, Harvard University, Cambridge, MA 02138, USA; 2 Harvard Forest, Harvard University, Petersham, MA 01366, USA; 3 European Organization for Nuclear Research (CERN), 1217 Meyrin, Switzerland; 4 Cavendish Laboratory, University of Cambridge, Cambridge CB3 0HE, UK; 5 Institute for Quantitative Social Science, Harvard University, Cambridge, MA 02138, USA

**Keywords:** Research data, Research management

## Abstract

In the last few decades, data-driven methods have come to dominate many fields of scientific inquiry. Open data and open-source software have enabled the rapid implementation of novel methods to manage and analyze the growing flood of data. However, it has become apparent that many scientific fields exhibit distressingly low rates of reproducibility. Although there are many dimensions to this issue, we believe that there is a lack of formalism used when describing end-to-end published results, from the data source to the analysis to the final published results. Even when authors do their best to make their research and data accessible, this lack of formalism reduces the clarity and efficiency of reporting, which contributes to issues of reproducibility. Data provenance aids both reproducibility through *systematic* and *formal* records of the relationships among data sources, processes, datasets, publications and researchers.

## Reproducibility

The success and power of science depends on the transparency and validation of its findings. However, issues with reproducibility have surfaced across a broad swath of scientific disciplines. Reports of such issues have emanated from fields ranging from the social sciences to physics and the life-sciences, including medicine^1^. Although the lack of reproducibility does not necessarily imply incorrect results^2^, it remains a worrisome issue. This comes at a time when the rate of scientific publication is increasing exponentially^3^. At the same time, the data and the processes that produce results are becoming more computationally demanding.

Reproducibility is the cornerstone of science, so it is imperative that we improve the quality and reliability of publications by going beyond the publication of results and data to making analytical processes, not only available, but more importantly, intelligible^4^. Too often, despite the best efforts of authors, transparency, adequate for the replication of computational processes, is elusive. We advocate open-data, open-source and *open-process*, which we define as the formal record of the workflow that produced a result. Changes to the pipeline that transforms raw data to results can lead to non-trivial differences in results, which are impossible to explain without sufficient reporting. For example, a re-examination of studies of carbon flux in forested ecosystems in the Amazon detected differences in estimates up to 140%, which could mean as much as 7 metric tons of carbon per year in an area roughly the size of a football field, resulting from small differences in analytical pipelines^5^. Also, seemingly simple details, such as the version of the initial (raw) data or versions of the analytical software programs, are often difficult to identify, and their absence makes replication of analyses impossible, even if the code is available.

## Provenance-aware research

We suggest that there is an opportunity for the implementation of formalized (following mathematical reasoning) methods for collecting analytical details. Such methods are essential for transparent scientific research as promoted by the data policies of many funding agencies, including the UK Engineering and Physical Sciences Research Council and the US National Science Foundation. Implementation of such methods can be achieved only by systematically capturing computational processes and presenting this information in a machine-actionable format. One possible solution is the use of *data provenance*, which is a formal representation of computational processes. The sheer quantity of data produced for analysis necessitates the use of complex computational tools for data management and analysis. This, in turn, creates a need for more precise descriptions of the origin of data, the transformations that have been applied to those data, and the implications of the results. Data provenance contains the information necessary to document these processes. However, it should be collected automatically in a manner amenable to automated reasoning, so that data origin, data processing, and results presentation are communicated to a user in an intelligible manner.

**Figure 1:**
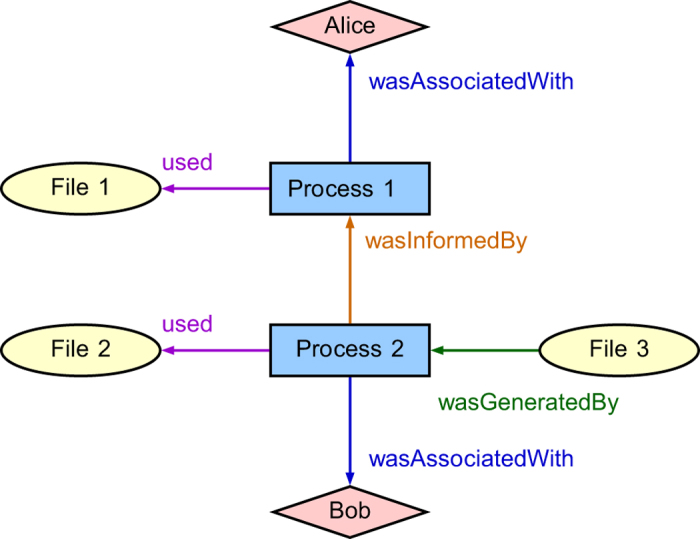
A simple W3C PROV-DM compliant provenance graph. In this example, two processes (Process1) and (Process 2), use the data from the inputs File 1 and File 2, respectively. The processes are associated respectively with the users Alice and Bob, respectively. Process 1 informed (transferred information to) Process 2, which generated the output File 3.

Provenance data is most frequently represented as a directed, acyclic graph. Interactions are recorded as a set of *edges* that relate data-items, transformations (computations), and persons or organizations associated with the data, all represented as *vertices* (see Fig. 1). This model has been standardized for interoperability by the World Wide Web Consortium (W3C) as the PROV data model (https://www.w3.org/TR/prov-dm/). While metadata standards with a related purpose have emerged in various fields (e.g. ISA-Tab in the biomedical space), the emergence of a common standard is important to avoid duplication of effort and to encourage interdisciplinary collaboration. This vision seems to be shared by part of the community as, for example, tools are being developed to convert between the previously mentioned ISA-Tab format and the W3C PROV standard model (see http://isa-tools.github.io/linkedISA/). Work is also in progress to support the upload of PROV-formatted provenance data on the Dataverse open-source repository platform. While no standard is perfect, we see the adoption of *a* common standard as a necessary step.

Exploiting data provenance is a multi-stage process. First, it involves the *capture* of data provenance during code execution. Next, the provenance must be *stored* in an efficient manner. Last, the provenance is queried and analysed either by machines (algorithmically) or by humans, most frequently through visualization.

Provenance capture can be divided into two broad categories: observed and disclosed^6^. *Disclosed* provenance consists of modifying an existing application so that it publishes the provenance resulting from its execution. One example of disclosed provenance is the Earth System Science Workbench^7^, used to process satellite imagery. *Observed* provenance consists of modifying the system on top of which the computation runs, so that it systematically and automatically records how data are generated. PASS^8^, which captures provenance in the operating system, is an observed provenance system. It produces a record of the execution of unmodified programs that run on top of it. The tension between these two approaches lies between in-depth domain specific knowledge for disclosed provenance and systematic, ubiquitous capture for observed provenance. PASS v2^9^ was the first system to allow both approaches to be used simultaneously. PASS pre-dates the W3C PROV standard by a few years; however, recent efforts for a modernised implementation of a similar concept, which adopts current best practices, is available online under an open-source licence (see http://camflow.org/).

A second aspect of provenance management is its storage. Numerous systems have been developed over the years to accomplish this. Some are domain-specific, whereas others have been intended for more general application. For example, the Core Provenance Library^10^ provides an interface between provenance generating applications and various database back-ends (a W3C PROV conforming open-source implementation is available at https://github.com/jacksonokuhn/prov-cpl). It enables the integration of provenance information from diverse sources into a coherent whole. Other issues, such as the scale of provenance generated by large scale systems, are being addressed using Big Data storage^11^.

The last aspect concerns the analysis, query and use of the provenance data. For example, visualization tools that present provenance data in an intelligible manner have been created by projects, such as Orbiter^12^ or VisTrails^13^. Another use of provenance is to render the analytical process transparent. By examining provenance records, one can learn how a team went from the raw collected data to the published results. Provenance can be seen in this context as a way to share this knowledge. Tools such as ReproZip^14^ have been built to automatically reproduce computational environments. Others have envisioned using these data to produce executable papers^15^ to allow readers and reviewers to repeat a computational experiment or conduct related experiments. Additionally, they can be used to verify a claim or test new hypotheses with less engineering effort. Examples of executable papers from the Association for Computing Machinery (a leading Computer Science publisher) Special Interest Group on Management of Data 2008 to 2011 conferences are available online http://event.cwi.nl/SIGMOD-RWE/. A maintained and updated list of existing provenance tools is available at https://projects.iq.harvard.edu/provenance-at-harvard.

## Data pipelines from particles to ecosystems

**Figure 2:**
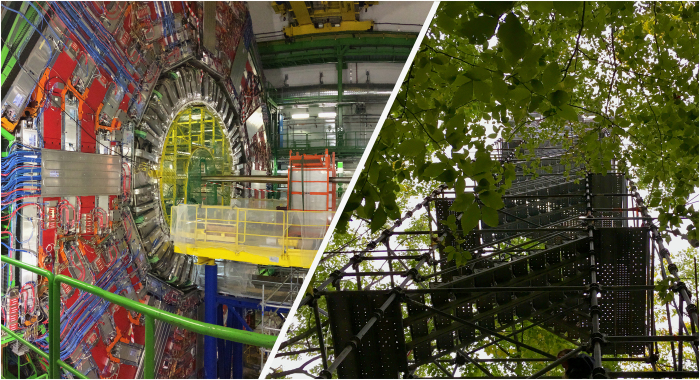
Research teams across the sciences are integrating data provenance methods into their research practices in response to increases in computational demands. On the left: (Photo Credit: A. Trisovic) The Compact Muon Solenoid (CMS) experiment at CERN during the technical stop in February 2017. On the right: (Photo Credit: M.K. Lau) One of several research towers used for ecological data collection at Harvard Forest. In addition to providing infrastructure for researchers to view the forest at multiple levels in the forest canopy, many instruments for automated observations, such as wind speed, CO_2_ flux, and leaf phenology, are placed on these towers. Data are relayed to a controlling computer via a wireless network.

As computer scientists have been developing tools to collect provenance, many fields are dealing with an explosion of data and software and the ensuing impacts on transparency and reproducibility^1^. Although there are field-specific issues that cannot be addressed by any single set of recommendations for transparency, the common issue is the need for a sharable record of computation. As we suggest above, a significant part of this challenge can be addressed across all fields via automated capture of formalized data provenance. To illustrate this, we have selected two case studies from our personal experiences, of research conducted at vastly different scales of inquiry: particle physics and ecology (see Fig. 2).

The European Organization for Nuclear Research (CERN) operates one of the world’s largest and most complex scientific instruments: the Large Hadron Collider (LHC). The LHC accelerates and collides protons and heavy ions to measure properties of elementary particles. By recreating the conditions that existed moments after the Big Bang inside the LHC, the physicists hope to discover how the early Universe evolved. At the other end of the spectrum of physical organization is Harvard Forest (HF), an ecological research site composed of over 1,600 hectares of forest and facilities for ecological education and research. Founded in 1,907, researchers have been actively collecting data at HF for over a hundred years. Data collected by researchers have historically focused on the abundance and distribution of species (trees and understory plants).

Both CERN and HF are experiencing rapid increases in computational demands. The lifetime of a CERN experiment is several decades, including active runs, periods of maintenance and system upgrades. The experimental components (particle detectors) are improved yearly, with the major advancements taking place every several years. Those changes modify both hardware and software resulting in changes in the data, which need to be documented^16^. The amount of data produced (up to 40 TBs^−1^) at an LHC experiment is impossible to store due to technological limitations. Data streams are filtered through a constantly improving selection system to extract information of scientifically significant particle decays^17^. The context of the measurements provided by the data provenance is crucial to the successful interpretation and analysis of the data themselves. For example, small changes in the experimental settings can bias the data, which can skew measurements. Furthermore, the energy at which the protons collide has been increasing over time, and has resulted in incompatibilities between data collected at different points in time^16^. Thus, details, such as what selection was being performed on the collision data and the detector conditions, directly impact what was being recorded during a LHC run.

At HF the volume of data and computational sophistication of studies have increased dramatically in recent years. Several study areas are contributing to this. Landscape-scale studies^18^, ecological genomics^19^, ecological simulations^20^, and sensor networks^21^ all produce large amounts of data that can be orders of magnitude greater in size than what was historically collected by ecologists. In addition to data volume, the diffuse nature of data collection, via field-based instruments (streamflow sensors^22^ and phenology cameras^23^), has lead to the removal of direct human observation from the data stream. Lastly, in addition to data driven issues, both for the purpose of data analysis and simply handling large quantities of data, ecologists have begun to produce a large amount of software with a proper version control system not always in place.

Currently, both CERN and HF are actively working to integrate capture and utilization of data provenance at different levels of completeness and formalization. At CERN, due to the extraordinary volume, the data are optimized and transformed to include particle identification and particle track reconstruction before they become available to physicists. The experiments at CERN capture data provenance that includes detector and beam conditions, selection system settings, and software used in data transformation and optimization^17^. All data from research activities at HF are curated in the Harvard Forest Data Archive, which has operated for nearly 30 years, guided by the site’s participation in two long-term U.S. National Science Foundation (NSF) programs: the Long Term Ecological Research (LTER) network and the National Earth Observatory Network (NEON). Although all projects submitted to the repository must adhere to the archive’s guidelines (largely determined by the LTER and NEON requirements), the submission format of the archive is flexible, and researchers are able to submit formalized provenance files as a part of their projects. However, this is currently not required nor is it generally done by researchers^22^.

These two examples, CERN and HF, provide a window into the utility of data provenance from tracking sub-atomic particles to recording the dynamics of whole ecosystems of interacting organisms. We find, regardless of the apparent differences between particle physics and ecology, that there is a common thread that spans these distinctions: the imperative to generate research that is intelligible to other investigators and to those that conducted it in the first place^24,25^. Ecology and physics, as well as all other scientific disciplines, have had methods for communicating findings and determining the veracity of data, such as notebooks, peer-review, metadata and culture (researcher, laboratory or institutional esteem); however, the current computationally-driven nature of these fields both necessitates and enables new ways to provide useful information in this regard.

## Conclusion & Discussion

Research reproducibility affects many fields. We suggest that providing access to data and source code are only the first two of many steps. Data-provenance formalizes and contextualizes the relationships among publications, data and software artifacts. Furthermore, publishing provenance increases the quality of a publication by providing the complete context of data collection and transformation. For example, as provenance promotes comparison across publications, it aids in comprehension between interesting new results and errors in analyses. Tools are being actively developed to help scientists capture those data in an unobtrusive manner with little disruption to their workflows.

The scientific community has an important, active part to play in how these tools are developed and deployed. As exemplified by CERN and Harvard Forest, scientists in a broad array of scientific discpilines are recognizing the need and utility of provenance capture tools. However, integrating the necessary technology requires broad cultural shifts that extend beyond disciplinary boundaries. Scientists, publishers, and educators must push for further transparency and formalism when describing computational analysis. Many existing open-data repositories host references to scripts—not to mention the open-source community with its own venues—while some, including Dataverse (http://dataverse.org/) and DataOne (http://dataone.org/), have been working on deploying solutions to support the contribution of data provenance alongside datasets and analytical scripts.

If data provenance becomes a well-established convention, eventually the provenance metadata associated with each dataset will provide the *complete* data record. Such a record enables data users to give credit to both the authors of a referenced dataset as well as all the contributors of datasets and software from which the data were derived. As a result, this provides incentives for researchers to share resources (data, code, and process) as it will increase the visibility and recognition of their work.

## Additional Information

**How to cite this article:** Pasquier, T. *et al.* If these data could talk. *Sci. Data* 4:170114 doi: 10.1038/sdata.2017.114 (2017).
